# GpDSR7, a Novel E3 Ubiquitin Ligase Gene in *Grimmia pilifera* Is Involved in Tolerance to Drought Stress in *Arabidopsis*

**DOI:** 10.1371/journal.pone.0155455

**Published:** 2016-05-26

**Authors:** Mengmeng Li, Yihao Li, Junyi Zhao, Hai Liu, Shenghua Jia, Jie Li, Heping Zhao, Shengcheng Han, Yingdian Wang

**Affiliations:** Beijing Key Laboratory of Gene Resource and Molecular Development, College of Life Sciences, Beijing Normal University, Beijing, China; Beijing Forestry University, CHINA

## Abstract

The growth and development of plants under drought stress depends mainly on the expression levels of various genes and modification of proteins. To clarify the molecular mechanism of drought-tolerance of plants, suppression subtractive hybridisation cDNA libraries were screened to identify drought-stress-responsive unigenes in *Grimmia pilifera*, and a novel E3 ubiquitin ligase gene, *GpDSR7*, was identified among the 240 responsive unigenes. *GpDSR7* expression was induced by various abiotic stresses, particularly by drought. GpDSR7 displayed E3 ubiquitin ligase activity *in vitro* and was exclusively localised on the ER membrane in *Arabidopsis* mesophyll protoplasts. *GpDSR7-*overexpressing transgenic *Arabidopsis* plants showed a high water content and survival ratio under drought stress. Moreover, the expression levels of some marker genes involved in drought stress were higher in the transgenic plants than in wild-type plants. These results suggest that GpDSR7, an E3 ubiquitin ligase, is involved in tolerance to drought stress at the protein modification level.

## Introduction

Plants are consistently exposed to various abiotic stresses during their life cycle, and they have evolved unique defence responses for survival [1]. Drought is a major abiotic stress that seriously affects plant growth and development; thus, it is responsible for marked reductions in crop yield [2]. Therefore, elucidating the mechanism that regulates drought resistance is important for understanding the balance between plant development and resistance, as well as providing guidance for agricultural improvement.

When subjected to drought stress, plants adapt by triggering a network of signalling events [3] and altering the expression of a large number of genes involved in biochemical, cellular, and physiological processes [4]. Drought-induced genes are generally classified into two categories. One category comprises genes that function mainly in protecting plants against drought stress (functional proteins). The other category comprises genes that encode regulatory proteins involved in regulation of the expression of stress-responsive genes [5]. Many high-throughput expressed sequence tag (EST) detection methods—such as cDNA microarray, differential display, suppression subtractive hybridisation (SSH), and RNAseq—have been employed to identify genes whose expression is altered in response to drought stress [6, 7]. However, the genetic mechanisms that regulate drought tolerance remain unclear. Therefore, the function of stress-inducible genes must be investigated to understand the molecular mechanisms of stress tolerance in plants.

In addition to the drought-induced regulation of gene expression, post-translational modification also plays an important role in the drought stress response [8]. Much evidence suggests that ubiquitination, mediated by specific E3 ligases, which usually induces degradation of the regulatory protein, plays a critical role in the abiotic stress response [9]. Several E1 enzymes, many E2s, and a large number of E3s have been identified in plants [10]. The wide diversity of E3s suggests that plants have evolved specific mechanisms to respond to individual environmental stresses [11]. Some E3s initially received attention because their mRNA transcript abundance was regulated by stress and/or ABA. For example, *SDIR1* (salt- and drought-induced RING finger 1), *XERICO* (encoding a RING-H2-type zinc-finger protein) in *Arabidopsis*, and Rma1H1 (RING membrane-anchor 1 homolog 1) in hot pepper are rapidly upregulated in response to salt and/or drought [12–14]. Additionally, SDIR1 and XERICO function as positive regulators in drought tolerance because they play roles in enhancing ABA-induced stomatal closure [12, 13, 15]. Furthermore, SDIR1 ubiquitinates SDIRIP1 (SDIR1-interacting protein 1), then regulates the expression of the downstream transcription factor gene *ABA-INSENSITIVE5* [15]. *Rma1H1* functions as a positive regulator in drought tolerance by mediating the ubiquitination of the water-channel protein PIP2;1 [14].

*Grimmia*, an ancient bryophyte descended from the earliest branching events, grows on exposed rock surfaces and can withstand extremely harsh environmental conditions [16–19]. It has been recorded for its remarkable vitality taken from herbarium specimens [19]. *Grimmia pilifera* P. Beauv., a *Grimmia* species, is distributed from temperate Asia to northern America [20], but its drought-stress-inducible genes have not been identified. In the present study, the gene expression profiles of *G*. *pilifera* under drought stress were determined by screening of suppression-subtractive hybridisation cDNA libraries. A hypothetical E3 ubiquitin ligase gene, *GpDSR7*, was found to be involved in the response to drought stress, and an *in vitro* ubiquitination assay showed that it functions as an E3 ubiquitin ligase and is localised to the plasma membrane. GpSDR7 production was induced by various abiotic stresses, and its overexpression conferred drought tolerance to *Arabidopsis* transgenic plants. These results suggest that *GpDSR7* encodes an E3 ubiquitin ligase that plays essential roles in regulating the response to drought tolerance through protein modification.

## Materials and Methods

### Plant materials and application of abiotic stresses

*Grimmia pilifera* P. Beauv. gametophytes were collected from Laoshan Mountain in Shandong Province, China. And we want to mention that no specific permissions were required for collecting Grimmia species in Laoshan Mountain in Shandong Province, China because the field where we collected the moss is not a national park or other protected area and and open to scientific research in China. we also want to say that Grimmis species is only a common Bryophyte plant, not a endangered or protected plant.

Each *G*. *pilifera* P. Beauv clump was carefully separated into single shoots and then washed thoroughly in running water to remove debris. Hydrated moss was obtained after a 24-h rehydration period [21]. For drought treatment, hydrated moss was desiccated rapidly in a desiccator containing activated silica gel particles (nearly 0% RH) [22]. To determine the water status, the RWC was calculated as follows: RWC = (FW − DW) / (TW − DW) × 100% [23], where FW is fresh weight (immediately weighed upon removal from the desiccator), DW is dry weight (desiccation at 80°C for 24 h until constant weight), and TW is turgid weight (24-h water saturation at 4°C in darkness). ABA- and salt-treated moss was obtained by incubating rehydrated gametophytes in 100 μM ABA (Sigma) or 100 mM NaCl solutions for the indicated times, respectively. Cold treatment was carried out by incubating moss samples at 0°C (on ice).

*Arabidopsis thaliana* ecotype Columbia was used as the WT in this study. Surface-sterilisation of seeds and conventional culture were performed as described previously [24].

### Construction of subtracted cDNA libraries

SSH cDNA libraries were constructed using a PCR-Select cDNA Subtraction Kit (Clontech) according to the manufacturer’s instruction. To obtain sufficient starting material for the SSH procedure, the cDNA fraction was amplified using the SMART PCR cDNA Synthesis Kit (Clontech). A mixture of 1 μg total RNA from material treated for 0.5 and 2.5 h was used as a tester, and control total RNA from hydrated gametophytes was used as a driver. Products of the secondary PCR from the forward and reverse subtraction were directly inserted into the pGEM-T vector (Promega) and transformed into *E*. *coli* JM109 cells. Recombinant clones were used to establish the subtracted cDNA library.

### Sequence analysis of the cloned ESTs

Randomly chosen clones were single-pass sequenced (Shanghai Sangong, China). The sequences obtained were fed into the VecScreen software (http://www.ncbi.nlm.nih.gov/VecScreen/VecScreen.html) to remove vector sequence contamination. All overlapping sequences were clustered into contigs using the Aligner software (CodonCode). Sequences of fewer than 150 nucleotides were excluded from clustering. Homology search and annotation were performed using the BLASTx software (cut off E-value 10^−5^) in the NCBI database. Functional classification of the unigenes was performed using the functional categories of *Arabidopsis* proteins (http://mips.gsf.de/projects/function) [25].

### Isolation and sequence analysis of GpDSR7

The 5’- and 3’-ends of *GpDSR7* were isolated using RACE reactions. Gene-specific primers (S1 Table) and a RACE cDNA amplification kit (Clontech) were used. The nested PCR products from each reaction were gel purified and cloned into the pGEM-T vector (Promega) for sequencing. After assembly of the 5’-RACE and 3’-RACE fragment sequences, the ORF of *GpDSR7* cDNA was amplified using specific primers (S1 Table) with the following program: 25 cycles at 94°C for 30 s, 56°C for 30 s, and 72°C for 1.5 min.

Domains of GpDSR7 were analysed using the SMART (http://smart.embl-heidelberg.de/) and Pfam (http://pfam.sanger.ac.uk/) databases. The deduced amino acid sequences of GpDSR7 and its orthologues were analysed and aligned using the ClustalW software (http://www.ebi.ac.uk/clustalw/).

### Subcellular localisation of GpDSR7

The full-length, TM domain, and TM domain deletion forms of *GpDSR7* were amplified by PCR using gene-specific primers (S1 Table). The PCR fragment was cloned into the pENTR^™^/D-TOPO entry vector (Invitrogen) and sub-cloned into the destination vector pMDC83 via LR recombination reaction (Invitrogen). Fresh onion (*Allium cepa* L) pieces were bombarded using a Bio-Rad He/1000 particle delivery system at 1,100 psi. Bombarded tissues were incubated in darkness in liquid MS medium for 24 h at 22°C. Images were obtained using an FV 300/IX70 inverted laser-scanning confocal microscope (Olympus) with excitation at 488 nm.

### Expression of recombinant proteins and in vitro self-ubiquitination analysis

The C-terminal TM truncated *GpDSR7*, *GpDSR7*^Δ^, was amplified by PCR using *BamH*I F and *Sal*I R primers, which introduces a 6× His tag at the C-terminal (S1 Table). The PCR products were inserted into the *BamH*I and *Sal*I sites of the pGEX-6p-1 vector (Amersham). Mutants of *GpDSR7*^Δ^ (C224S, C242S, and H250Y) were generated using the PCR overlap extension method [26]. The pairs of mutagenic oligonucleotide primers for site-directed mutagenesis are shown in S1 Table. The WT and mutant recombinant GST-GpDSR7^Δ^ fusion proteins were expressed in *E*. *coli* Rosetta (DE3) cells and purified using a nickel-nitrilotriacetic acid agarose matrix.

For the E3 ubiquitin ligase activity assay, approximately 500 ng of purified GST-GpDSR7^Δ^ fusion protein were mixed with 100 ng human E1 (Boston Biochem), 200 ng human E2 UBCH5C (Boston Biochem), and 5 μg ubiquitin-myc (Boston Biochem). The reactions were performed in buffer containing 50 mM Tris-HCl (pH 7.5), 5 mM MgCl_2_, 2 mM ATP, and 2 mM DTT and incubated for 1 h at 30°C. The reactions were stopped by adding SDS loading buffer and boiling at 100°C for 5 min. The reaction products were separated in 8% SDS-PAGE gels and blotted onto polyvinylidene difluoride membranes (Millipore). Immunoblotting was carried out using an anti-GST antibody (Sigma) or anti-Myc antibody (Santa Cruz).

### Generation of transgenic Arabidopsis plants and phenotypic analysis

The full-length cDNA of *GpDSR7* without the stop codon was amplified using the primers GpDSR7 pENTR F and pENTR R. The PCR product was introduced by BP recombination into the pENTR/D-TOPO entry vector (Invitrogen) and translocated into the pEarlygate103 destination vector. *35S*:*GpDSR7* transgenic *Arabidopsis* was generated as described by Ko *et al*. [13]. mRNA and protein levels were determined by RT-PCR and immunoblotting, respectively.

For evaluation of drought resistance, 1-week-old seedlings were transplanted to soil for 2 weeks under standard growth conditions. The plants were then subjected to sustained drought by ceasing watering for the indicated times, followed by re-watering for 7 days. Survival rates were calculated 7 days later. The water loss assay was performed as described by Lee [14]. Leaf water loss was monitored as a percentage of that at the initial time. For gene expression analysis, 2-week-old seedlings grown in agar plates were transferred onto Whatman 3-mm filter paper and subjected to drought treatment.

### RNA extraction, RT-PCR, and real-time RT-PCR analyses

Total RNA was extracted from *G*. *pilifera* harvested at 0.00, 0.25, 0.50, 1.00, and 2.50 h after drought treatment using an RNeasy Plant Mini Kit (Qiagen). DNA contamination was removed using a Free DNase Set (Qiagen). First-strand cDNA synthesis was performed using the M-MLV reverse transcriptase (Promega). *Actin* was used as the internal control. The primer sequences and optimal PCR cycles are shown in S2 Table.

To determine the expression levels of stress-responsive genes in *Arabidopsis* under drought stress, quantitative real-time PCR analysis was performed. TRIzol reagent (Invitrogen) was used to extract total RNA. First-strand cDNA was synthesised using a QuantiTect reverse transcription kit (Qiagen). Real-time PCR was performed using SYBR Green qPCR Mix (Applied Biosystems) on an ABI 7500HT System (Applied Biosystems). Primers are listed in S3 Table. Data were normalised to the glyceraldehyde-3-phosphate dehydrogenase (GAPDH) mRNA levels.

## Results

### Identification of drought-stress-responsive genes in G. pilifera

**Fig 1:**
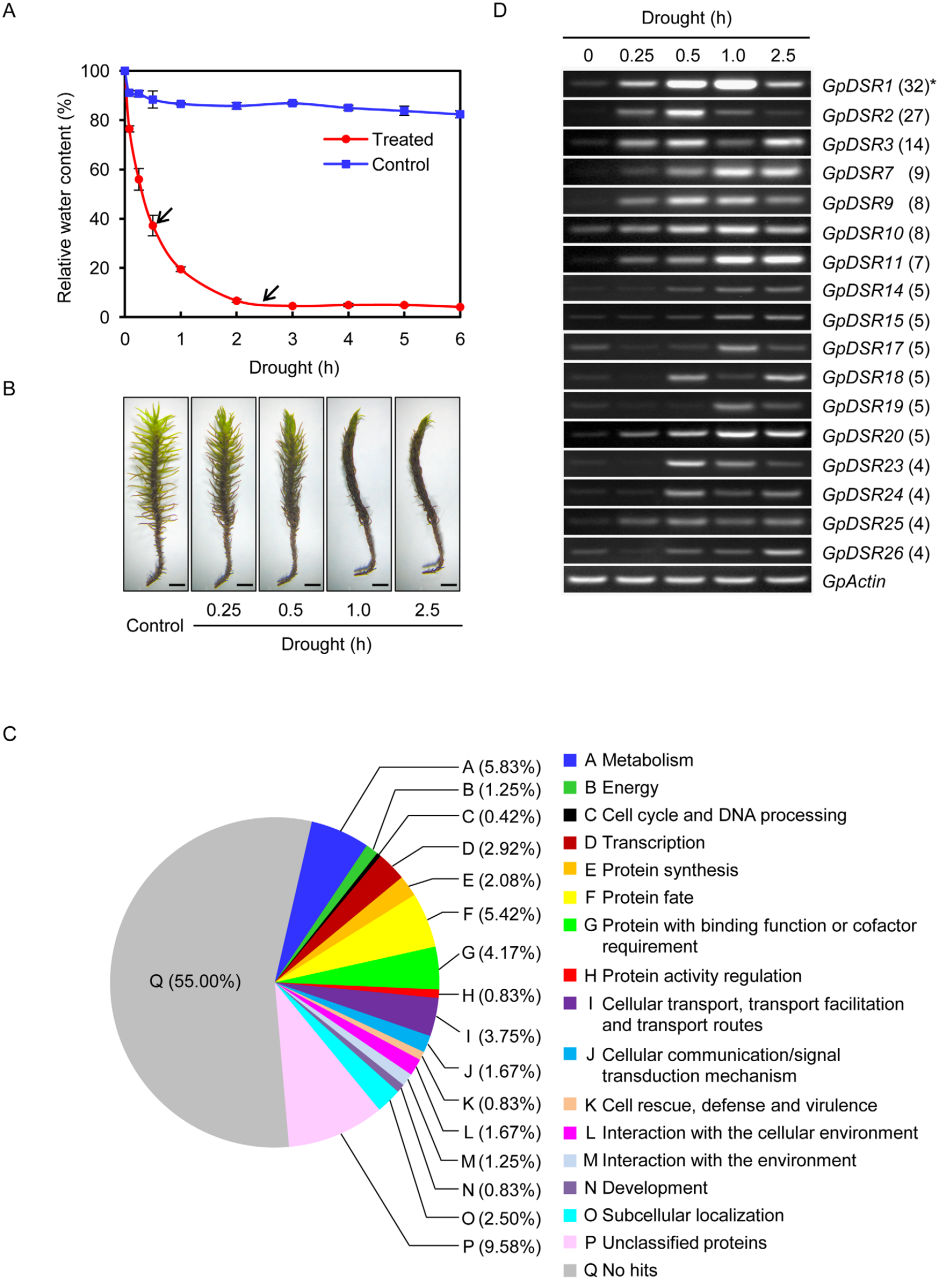
General summarisation of SSH cDNA libraries in *G*. *pilifera*. (A) Relative water contents were measured to determine the water status of dehydrated gametophytes. Arrows indicate 0.5- and 2.5-h treatments, which were selected for SSH cDNA library construction. Bars are means ± SD of five biological replicates. (B) Morphologies of *G*. *pilifera* gametophytes under fully hydrated and dehydrated conditions. Bar = 2 mm. (C) Percentage distribution of unigenes categorised into functional classes. (D) Expression patterns of several drought-responsive genes identified by SSH. Total RNA was extracted from *G*. *pilifera* at 0.00, 0.25, 0.50, 1.00, and 2.50 h after drought stress treatment and analysed by qRT-PCR using gene-specific primers. *Actin* was used as the internal control. *Numbers in parentheses are numbers of EST fragments of the *GpDSR* genes in the SSH library.

To determine the water status of samples and select appropriate stress conditions for constructing the subtracted cDNA library, the relative water content (RWC) was monitored during dehydration treatment. As shown in Fig 1A, the RWC of stressed plants decreased rapidly, reaching approximately 5% after 2.5 h, and then remaining stable at 4% throughout the dehydration treatment. Moreover, the morphology of *G*. *pilifera* is shown in Fig 1B. Early and late drought responses may occur at 0.5 and 2.5 h after dehydration, respectively; thus, dehydrated gametophytes at these time points were used to construct the subtractive hybridisation library.

Only the forward SSH cDNA library was used in further experiments because it represented genes either upregulated or specifically expressed in response to drought. After cloning and sequencing, 574 readable ESTs were obtained, which represented 240 unigenes containing 114 singletons and 126 contigs (S4 Table). The corresponding proteins were sorted into various groups, including 15 functional categories (A–O, Fig 1C), unclassified (unknown and unnamed) proteins (P), and proteins with no matches in the database (Q). As shown in Fig 1C, the genes encoding proteins responsible for metabolism (A, 5.83%), protein fate (F, 5.42%), and protein with binding function or cofactor requirement (G, 4.17%) may play important roles in the drought-tolerance mechanisms of *G*. *pilifera*.

Based on the EST abundance, 31 unigenes assembled with more than 4 ESTs were referred to as *G*. *p**ilifera* drought stress responsive genes (*GpDSRs*) (Table 1). To verify *GpDSR* expression patterns, 17 unigenes were subjected to RT-PCR analysis (Fig 1D). Most of the genes were induced under drought stress, although their levels of induction differed. In addition, the expression levels of unigenes matched their EST abundance (or redundancy) because those genes with high EST redundancy showed greater intensities than those with low EST redundancy (Fig 1D).

**Table 1:** Unigenes assembled with more than four ESTs annotated by sequence similarities.

Unigene^a^	No. of EST fragments	Length (bp)	Annotation^b^	Accession No.^c^	Functional categorisation^d^
*GpDSR1*	32	857	Chlorophyll a-b binding protein CP26	At4g10340	B
*GpDSR2*	27	1005	Hyperosmolality-gated calcium-permeable channel	At4g04340	P
*GpDSR3*	14	937	RING/U-box superfamily protein	At1g78420	G
*GpDSR4*	13	957	–	–	Q
*GpDSR5*	10	819	–	–	Q
*GpDSR6*	9	1096	Hyperosmolality-gated calcium-permeable channel	At4g04340	P
GpDSR7*	9	1049	RING/U-box domain-containing protein	At5g60580	G
*GpDSR8*	9	953	–	–	Q
*GpDSR9*	8	746	Aspartyl protease family protein	At2g36670	F
*GpDSR10*	8	426	Ribulose bisphosphate carboxylase small chain	At1g67090	A
*GpDSR11*	7	1024	Trehalose-6-phosphate synthase	At4g17770	A
*GpDSR12*	6	740	–	–	Q
*GpDSR13*	6	680	–	–	Q
*GpDSR14*	5	810	Phospholipase D	At4g35790	J
*GpDSR15*	5	1006	Cationic amino acid transmembrane transporter	At5g04770	B
*GpDSR16*	5	1079	Hyperosmolality-gated calcium-permeable channel	At4g04340	P
*GpDSR17*	5	1186	Erythronate-4-phosphate dehydrogenase family protein	At1g75180	P
*GpDSR18*	5	1071	EXS family protein	At5g35730	P
*GpDSR19*	5	567	Sodium/hydrogen antiporter	At5g27150	L
*GpDSR20*	5	963	TLP3B TIR1-like auxin receptor protein	At2g39940	J
*GpDSR21*	5	809	–	–	Q
*GpDSR22*	5	153	–		Q
*GpDSR23*	4	835	Allene oxide synthase	At5g42650	K
*GpDSR24*	4	917	DNA methyltransferase 1-associated protein	At2g47210	D
*GpDSR25*	4	1026	Glucose-6-phosphate/phosphate translocator- like protein	At5g46110	I
*GpDSR26*	4	696	Photosystem I reaction centre subunit XI	At4g12800	P
*GpDSR27*	4	461	Uncharacterised protein	At2g44670	P
*GpDSR28*	4	690	–	–	Q
*GpDSR29*	4	551	–	–	Q
*GpDSR30*	4	339	–	–	Q
*GpDSR31*	4	391	–	–	Q

^a^ *GpDSRs* refers to *G**rimma* *p**ilifera* drought stress-responsive genes.

^b^ Annotation corresponded to the hit with the highest score in an NCBI BLASTx search.

^c^ Locus numbers indicate the database sequences used for comparison with the *Arabidopsis* sequences.

^d^ Functional categories were generated according to MIPS, as shown in Fig 1.

### Characterisation of the full-length cDNA of GpDSR7

**Fig 2:**
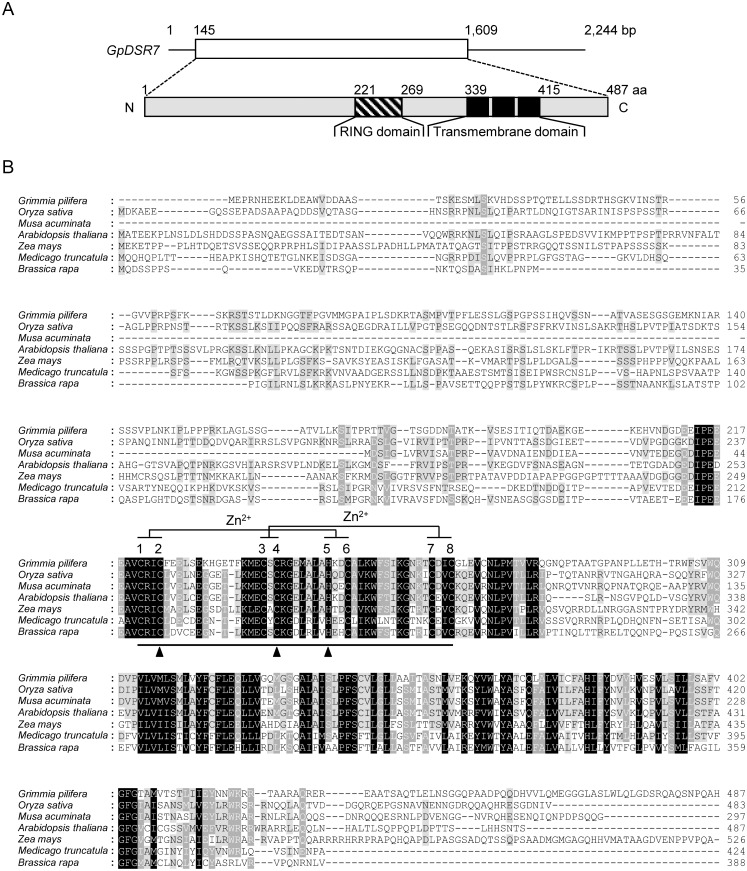
Sequence analysis of GpDSR7. (A) Schematic structure of the *GpDSR7* cDNA and predicted GpDSR7 protein. Hatched bar indicates the RING (Really Interesting New Gene) motif, and dark bar indicates the C-terminal putative TM domain. (B) Comparison of the derived amino acid sequence of GpDSR7 with its homologues from other species, including rice (Os06g0677300), *Musa acuminata* (ABF69983.1), *Arabidopsis* (At5g60580), maize (NP_001148132.1), *Medicago truncatula* (XP_003594026.1), and *Brassica rapa* (ADK63404.1). Solid line denotes the RING motif, and conserved metal ligand positions are indicated by numbered cysteine (C) and histidine (H) residues. Triangles indicate the sites of mutations (C224S, C242S, and H250Y).

We identified a broad spectrum of partial cDNA clones in *G*. *pilifera* that were induced by drought. *GpDSR7* encodes a polypeptide that contains a RING/U-box domain-containing protein, and its mRNA level is markedly induced by drought stress (Fig 1D). GpDSR7 is predicted to be of 52.5 kDa with a calculated pI of 5.62. Fig 2 shows the nucleotide and protein sequences of GpDSR7. It possesses three putative transmembrane (TM) domains near the C-terminal, suggesting that it to be membrane-associated (Fig 2A). GpDSR7 also contains a C4H3-type RING domain from residues 221 to 269, which comprises seven conserved cysteines and a histidine (Fig 2B). A database search indicated that the C4H3 domain of GpDSR7 shows 38% to 57% identity with the corresponding domain in other plants (Fig 2B).

### Expression patterns of GpDSR7 in response to various abiotic stresses

**Fig 3:**
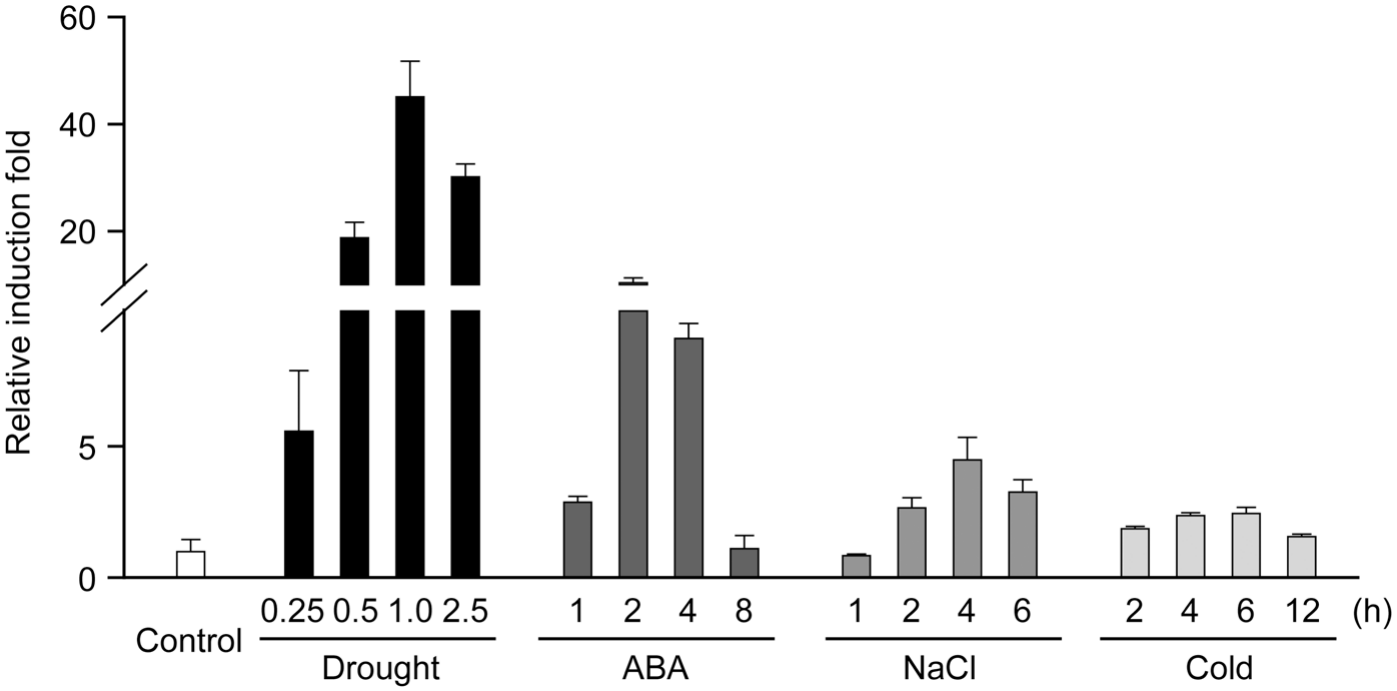
Expression of *GpDSR7* in response to various abiotic-related stresses in *G*. *pilifera*. Rehydrated *G*. *pilifera* gametophytes were subjected to drought, ABA, high salinity, or low-temperature stress. Expression patterns of *GpDSR7* were determined by qRT-PCR. *GpDSR7* expression without treatment was set as 1.0. *18S rRNA* was used as the internal control.

In order to confirm whether the expression of *GpDSR7* was induced by other abiotic stresses as well as drought, we have performed qRT-PCR experiments to detect the expression pattern of *GpDSR7*. As shown in Fig 3, the *GpDSR7* transcript level increased dramatically upon exposure to drought, and then declined slightly after severe water loss (RWC 4%). Interestingly, the expression pattern of *GpDSR7* following application of exogenous ABA is similar to that with drought treatment, however the drought is more effective to trigger the transcription of *GpDSR7*. Likewise, salt stress enhanced *GpDSR7* expression, albeit to a lesser degree than drought stress. *GpDSR7* accumulation was not obviously affected by low temperature. These results suggest that *GpDSR7* is involved in the response to drought stress of *G*. *pilifera*.

### GpDSR7 functions as a RING E3 Ub ligase

**Fig 4:**
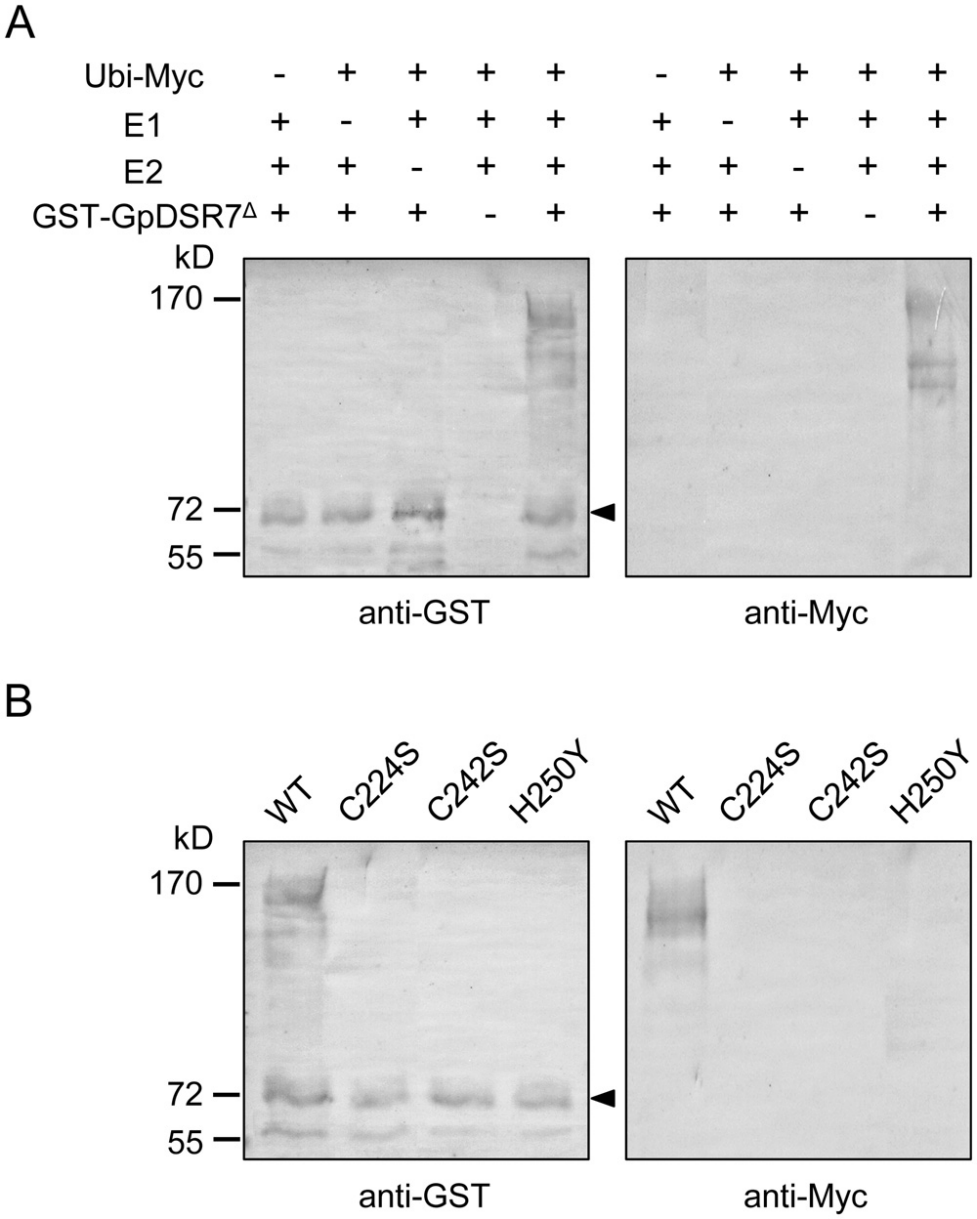
*In vitro* self-ubiquitination assay of GpDSR7. The C-terminal TM domain truncated form of GpDSR7 was defined as GpDSR7^Δ^. (A) GST-tagged GpDSR7^Δ^ fusion protein was assayed for E3 ubiquitin ligase activity in the presence of E1, E2, and Ubi-Myc. Ubiquitinated proteins were detected by western blot analysis using an anti-GST antibody (left panel) and anti-Myc antibody (right panel). Triangles indicate non-ubiquitinated GST-GpDSR7^Δ^. (B) Purified WT GST-GpDSR7^Δ^ and mutant proteins were used in the E3 Ub ligase enzyme assay.

Previous research has shown that many RING-containing proteins function as E3 Ub ligases. To examine whether GpDSR7 possesses E3 Ub ligase activity, the TM domain-truncated form of GpDSR7 (GpDSR7^Δ^) was expressed in *Escherichia coli* as a GST-fusion protein. The purified GST-GpDSR7^Δ^ protein was incubated at 30°C in the presence or absence of myc-tagged ubiquitin (ubi-myc), human E1, and human UBCH5c (E2) for 1 h. As shown in Fig 4A, GST-GpDSR7^Δ^ gave rise to high-molecular-mass ubiquitinated smear ladders, while no ubiquitinated signals were detected in the absence of E1, E2, ATP, or Ub. Additionally, we constructed single amino acid substitution mutants of GST-GpRSD7^Δ^, in which Cys^224^, Cys^242^, and His^250^ residues in the RING domain were replaced with Ser, Ser, and Tyr, respectively (Fig 2B). In contrast to the wild type (WT), all of the mutants lost their Ub ligase activity almost completely (Fig 4B). These data indicate that *GpDSR7* encodes a functional E3 ubiquitin ligase and that the intact RING domain is essential for its activity.

### GpDSD7 localises to the endoplasmic reticulum (ER) membrane

**Fig 5:**
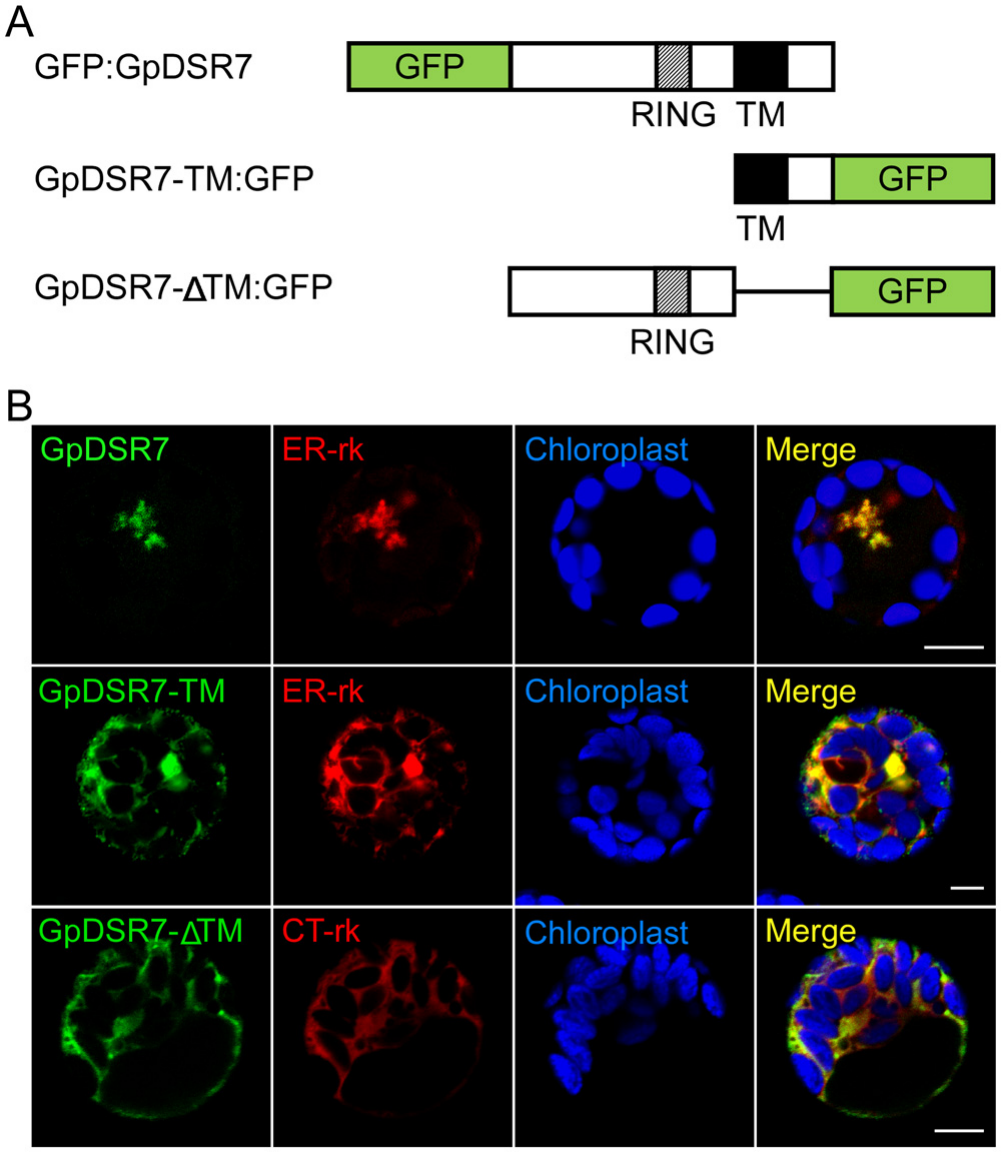
Subcellular localisation of GpDSR7 in *Arabidopsis* mesophyll protoplasts. (A) Schematic of GpDSR7 and constructs used in the subcellular localisation assay. Lines indicate deleted regions. TM, transmembrane domain; RING, RING finger domain. (B) The fusion constructs GpDSR7-GFP, GpDSR7-TM-GFP and GpDSR7-ΔTM-GFP were transiently cotransformed with mCherry-labelled ER marker or cytoplasm (CT) marker into *Arabidopsis* mesophyll cells. All images were obtained from one optic section. Scale bars are equivalent to 10 μm.

To investigate the subcellular localisation of GpDSR7, the full length and truncated forms of GpDSR7 fused with Green fluorescent protein (GFP) were generated (Fig 5A) and transiently co-transformed with mCherry-labelled marker constructs in the *Arabidopsis* mesophyll protoplasts. The full-size GpDSR7 was dispersed in the cytoplasm and mainly co-localised with the ER marker. And the GpDSR7-TM fluorescence signal was also detected at the ER membrane, while the GpDSR7-ΔTM:GFP signal was uniformly distributed in the cytosol (Fig 5B). These results suggest that the TM domain of GpDSR7 is essential for its ER membrane localisation.

### Overexpression of GpDSR7 confers drought tolerance in Arabidopsis

**Fig 6:**
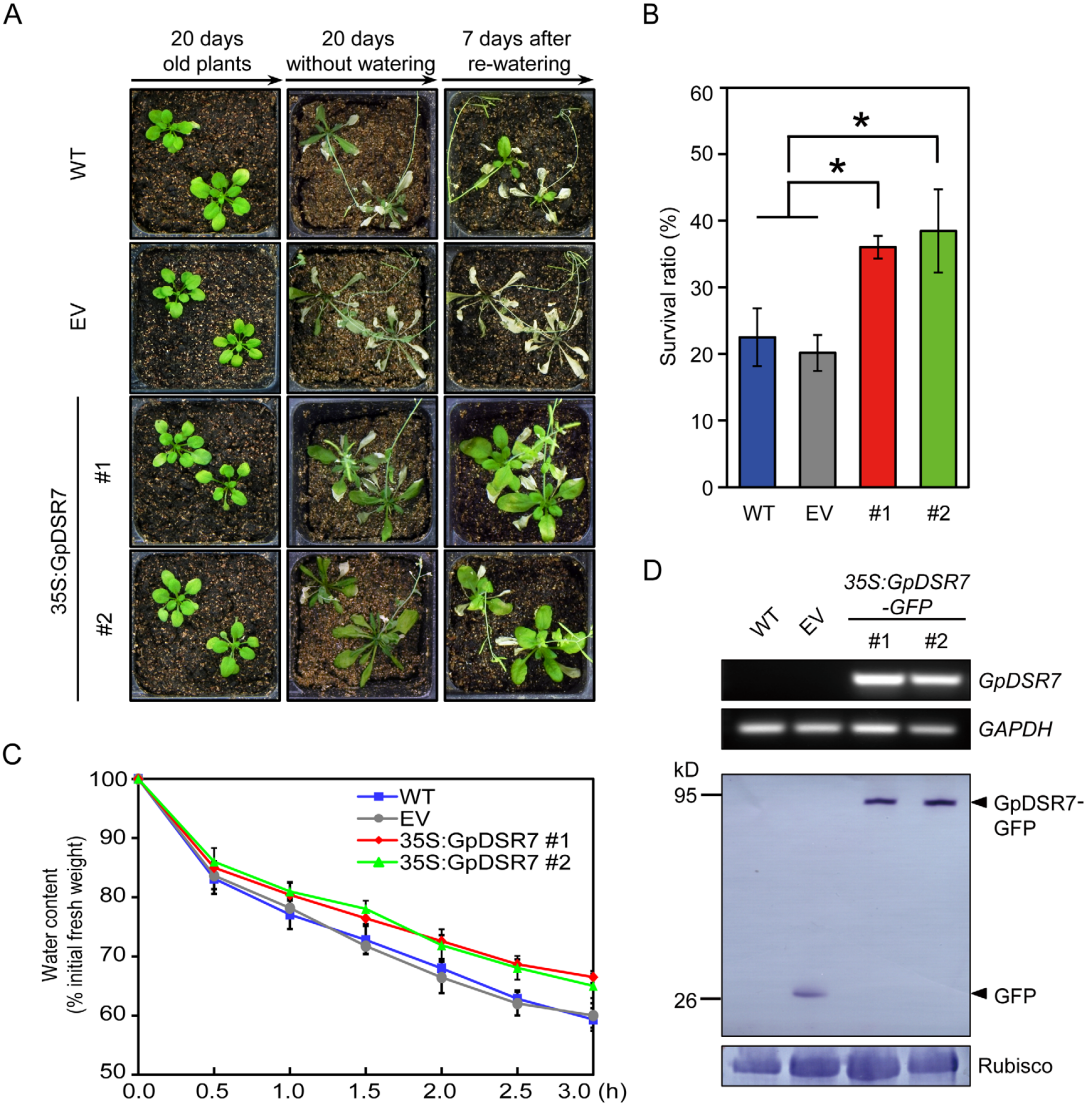
Drought response of *35S*:*GpDSR7* over-expressed transgenic *Arabidospsis* plants. (A) Seven-day-old seedlings were transferred to soil for a further 2 weeks of normal growth (left panel), subjected to progressive drought by withholding water for 20 days (middle panel), and then re-watered for 7 days (right panel). (B) Survival rate of plants after re-watering (n > 40). Asterisks indicate significant differences determined by Student’s *t* test (* 0.01 < P < 0.05). (C) Leaves of 20-day-old plants were excised and weighed at various time points after detachment. Values are means ± SD of three individual plants. (D) Two independent T3 transgenic lines #1 and #2 were subjected to RT-PCR (upper) and immunoblotting (lower) for detecting the expression of GpDSR7. We have used the wildtype (WT) and the plants tranformed with empty vector (EV) as the control.

Because *GpDSR7* is a drought-inducible gene, it may function in drought response. To evaluate this hypothesis, *GpDSR7* was overexpressed in *Arabidopsis* under the control of the CaMV 35S promoter (Fig 6D). Three-week-old *Arabidopsis* plants were grown under normal conditions before drought treatment. All plants without watered for 20 days exhibited wilt, although the *35S*:*GpDSR7* plants displayed less severe wilting. After re-watering for 7 days, most WT and plants transformed with empty vector were unable to recover and had a relatively low survival rate of 22.5%. However, the *GpDSR7*-overexpressing lines exhibited higher survival rates (36% for line #1 and 38.4% for line #2) and continued to grow (Fig 6A and 6B). To further evaluate the anti-drought abilities, rosette leaves were excised from plants and the water loss rates were determined. Decreases in fresh weights were measured over time (0–3 h). As shown in Fig 6C, the greatest rate of water loss occurred during the first 30 min after detachment. Subsequently, the water loss rates of detached rosette leaves from *35S*:*GpDSR7* plants were lower than those of WT plants (Fig 6C). Thus, we concluded that overexpression of *GpDSR7* conferred drought tolerance on transgenic *Arabidopsis*.

### GpDSR7 positively regulated the expression of stress-responsive genes in transgenic plants

**Fig 7:**
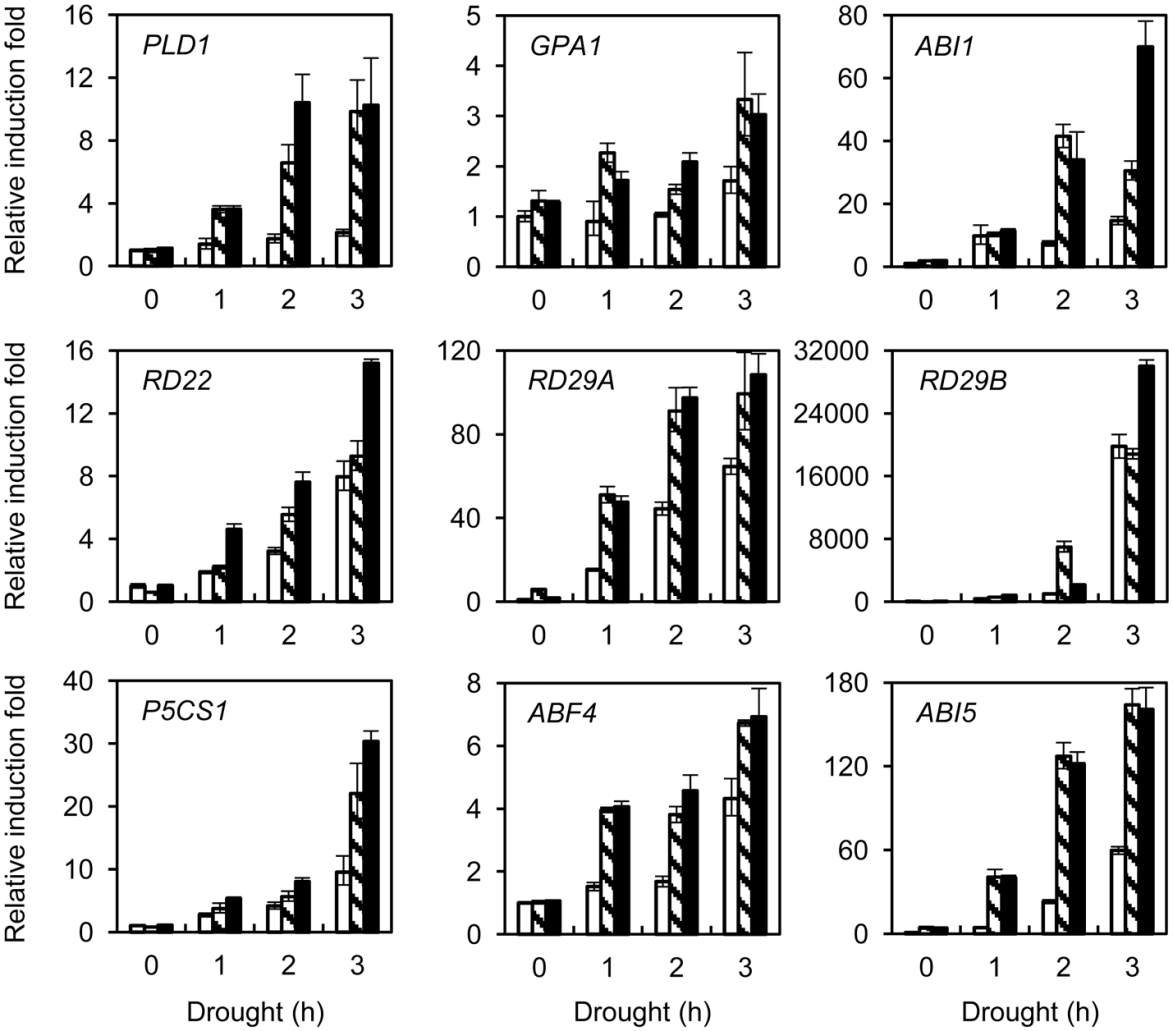
Expression of drought-stress-responsive genes in wild-type and *GpDSR7-*overexpressed *Arabidopsis*. Total RNA was extracted from control and drought-treated plants and the expression of several drought-responsive gene was analysed by qRT-PCR. The mean value of three technical replicates was normalised to the expression level of glyceraldehyde-3-phosphate dehydrogenase (GAPDH). Blank columns: control group; slash columns: #1 transgenic plants; black columns: #2 transgenic plants.

To determine whether GpDSR7 regulates genes downstream of drought and ABA signalling, qRT-PCR analysis was used to examine the expression of genes related to ABA-mediated stomatal closure, the drought response, and transcriptional regulation. *PLDα1* encodes phospholipase Dα, which regulates ABA-mediated stomatal closure through bifurcating interactions with protein phosphatase 2Cs and G proteins [27]. The result showed significant induction of *PLDα1*. The expression levels of *ABI1* (encodes the ABA-responsive protein phosphatase 2C) and *GPA1* (encodes the Gα subunit of heterotrimeric G protein) were also induced in the overexpressing plant lines (Fig 7). In addition, the drought-inducible genes *RD22*, *RD29A*, and *RD29B* were expressed at high levels in both control and transgenic plants under dehydration stress (Fig 7). Proline accumulation has been reported to function as a molecular chaperone that stabilises protein structure [28]. The expression of *P5CS1* (Δ^1^-pyrroline-5-carboxylate synthase 1), the rate-limiting enzyme in proline biosynthesis [29], was significantly higher in the transgenic than WT plants at the late stage of dehydration (Fig 7). Furthermore, we assessed the expression of two nuclear genes, *ABF4* and *ABI5*, which encode ABA-responsive basic leucine zipper (bZIP) transcription factors. Their mRNA levels were considerably higher in *35S*:*GpDSR7* transgenic plants than in WT plants (Fig 7). Taken together, these results suggest that GpDSR7 is involved in positive regulation of drought stress.

## Discussion

Using SSH technology, 240 drought-stress-related unigenes were identified in *G*. *pilifera*. Furthermore, the expression patterns of 17 *GpDSRs* were verified by RT-PCR (Fig 1D). The products of these *GpDSRs* can be divided into two categories: functional proteins that directly protect plants and regulatory proteins [5]. In the first group, *GpDSR1* encoded the putative chlorophyll a-b binding protein CP26 and was rapidly upregulated under drought stress. LHCB5 (also called CP26) has been proposed to regulate the transduction of light energy through the xanthophyll cycle to disperse high irradiance [30]. Three isoforms, *GpDSR2*, *GpDSR6*, and *GpDSR16*, encode the hyperosmolality-gated calcium-permeable channels (OSCAs), which were characterised as an osmosensor in *Arabidopsis* and involved in the perception of extracellular changes to trigger hyperosmolality-induced cytosolic Ca^2+^ increases [31]. Previous study showed that nine of eleven *OsOSCA* genes were down- or upregulated (<0.5 or>2) in responsive to osmotic-related abiotic stress in *Oryza sativa* [32]. GpDSR19, a putative Na^+^/H^+^ antiporter, is reportedly responsible for maintenance of ionic homeostasis because it plays roles in the transportation and compartmentalisation of iron [33, 34]. In addition, we detected several genes related to osmotic homeostasis. For example, trehalose-6-phosphate synthase (a homologue of GpDSR11) is responsible for trehalose synthesis [35]. It is hypothesised that trehalose, as a storage sugar, may promote formation of a protective glassy matrix that contributes to resistance to desiccation [36]. The second group of drought-responsive genes comprises those encoding regulatory proteins. For example, *GpDSR24* encodes a putative DNA methyltransferase 1-associated protein, which may be involved in transcriptional regulation [37]. *GpDSR3* and *GpDSR7* encode RING/U-box superfamily proteins, which belong to the E3 ligases and are likely involved in the ubiquitin-mediated 26S proteasome pathway. In the resurrection plant *Tortula ruralis*, the ubiquitin transcript level is reportedly sensitive to dehydration and rehydration [38]. *Grimmia pilifera*, similar to *T*. *ruralis*, usually undergoes rapid drying; thus, the ubiquitin-proteasome pathway may enhance the survival rate of plants under extreme drought conditions. In addition to functional proteins and regulators, a large number of proteins with no significant homology were detected (Fig 1C). This may be due to the lack of *G*. *pilifera* sequences in the NCBI database and the physiological state (i.e., desiccated material) of the samples used.

Recently, plant RING E3 ligases have attracted much attention because they play an important role in the perception and signal transduction of various internal (hormone) and external environmental signals [39]. Zhang *et al* showed that the endoplasmic reticulum membrane-localized E3 ligase SDIR1 (Salt- and drought-induced really interesting new gene finger1) plays a key role in ABA signaling, regulating ABA-related seed germination and the drought- and salt-stress response in *Arabidopsis* [12, 15]. In addition, SDIR1-Interacting Protein1 (SDIRIP1) functioned as a substrate of SDIR1 to negatively regulate ABA signaling by affecting the transcriptional expression of *ABI5*, which mediates the salt tolerence, but not drought tolerance [15]. These results indicated that other substrates of SDIR1 will present to regulat the response to drought stress in *Arabidopsis* [39, 40]. Four C3HC4-RING finger E3 ubiquitination ligases, *Arabidopsis* ABA-insensitive RING protein (AIRP1–4), were identified to be involved in regulating ABA-mediated stress responses [24, 41–43]. Over-expression of AIRP1, AIRP2 or AIRP3 reciprocally rescued ABA insensitivity and susceptibility to dehydration stress phenotypes of *airp1*, *airp2* and *airp3* mutant, suggesting that AIRP1, AIRP2 and AIRP3 play combinatory roles in ABA-mediated drought stress responses [24, 41, 42]. However, AIRP4 only responds to ABA at the post-germination stage, AIRP4 overexpression plants were hypersensitive to salt and osmotic stresses during seed germination, and showed drought avoidance compared with the wild-type and *airp4* mutant plants [43]. In this study, GpDSR3 and GpDSR7 were identified as putative E3 ligases based on a homology search. And GpDSR7 was verified as a RING E3 Ub ligase and its expression was upregulated dramatically under drought stress and ABA treatment, but less induced by NaCl and low temperature. So we have hetero-expressed GpDSR7 in *Arabidopsis* plants to further examine the function of GpDSR7.

Phenotypic analysis showed that the overexpression lines had enhanced tolerance to drought as indicated by the higher water content in detached leaves than in WT plants (Fig 6A and 6C). It was speculated that the enhanced drought tolerance of *GpDSR7-*overexpressing plants might be due to a low transpiration rate, which is regulated by stomata status. The expression levels of *PLD1*, *GPA1*, and *ABI1*, which are related to ABA-mediated stomatal closure, were upregulated in overexpression compared with WT plants (Fig 7). Moreover, *GpDSR7* induced the expression of genes downstream of ABA and/or other stress pathways. *RD29A*, *RD29B*, and *RD22* are induced by dehydration and ABA, and so elevated expression may improve plant tolerance to stresses [44]. In our study, *RD29A*, *RD29B*, and *RD22* were upregulated in both WT and transgenic plants under dehydration; indeed, the *RD29A* expression levels were significantly higher in the *35S*:*GpDSR7* line than in the WT. The promoter of RD29A contains two major *cis*-acting elements, ABRE and DRE, which function in ABA-dependent and -independent gene expression, respectively [5]. In addition, the expression levels of the functional gene *P5CS1* (Δ^1^-pyrroline-5-carboxylate synthetase1) and two regulatory genes *ABF4* (ABA-responsive element binding factor 4) and *ABI5* (ABA-insensitive 5) were higher in the transgenic lines than in WT plants. P5CS1 is the rate-limiting enzyme in proline synthesis, and its promoter region contains one DRE and five ABRE cis-elements. ABF4 and ABI5 encode ABA-responsive basic leucine zipper (bZIP) transcription factors [45], and their expression was induced by ABA. A study of *Arabidopsis SDIR1* and *AIRP1* indicated that they positively regulate ABA signalling and act upstream of bZIP transcription factors, such as ABI5, ABF3, and ABF4 [12, 15, 24]. We also found that there was no difference of the seed germination and post-germination growth in response to ABA between WT and overexpressed transgenic plants (data not shown). Therefore, GpDSR7 may act as a positive regulator of the expression of downstream stress-related genes by ABA-dependent and/or ABA-independent pathways during drought stress in transgenic *Arabidopsis*.

On the basis of previous studies and our results, we speculate that GpDSR7 functions as an E3 ligase that mediates the degradation of substrates. However, the detailed mechanism of action of GpDSR7 is not fully understood. GpDSR7 may function as a positive regulator and/or degrade unknown negative regulator(s), thereby activating the drought-signalling pathway. Therefore, GpDSR7 is involved in the drought-stress response, possibly by regulating stress-related proteins or expression of functional genes indirectly.

## Supporting Information

S1 Table Primers for PCR, cloning, and construction of vectors. (DOCX)

S2 Table Specific primers sequences for RT-PCR of *Grimmia pilifera*. (DOCX)

S3 Table Specific primers sequences for qRT-PCR expression analysis of drought stress-related genes in *Arabidopsis*. (DOCX)

S4 Table Summary on screening results of forward library ESTs cloned by SSH. (DOCX)
