# Supplementary material for: GpDSR7, a Novel E3 Ubiquitin Ligase Gene in Grimmia pilifera Is Involved in Tolerance to Drought Stress in Arabidopsis
Source: PLoS One. 2016 May 26;11(5):e0155455. doi: 10.1371/journal.pone.0155455 (PMC4882056; doi:10.1371/journal.pone.0155455)
Supplement: S1 Table — (DOCX) [file pone.0155455.s001.docx]

**S1 table. Primers for PCR, cloning, and construction of vectors.**

| Primer name | Forward | Reverse |
| --- | --- | --- |
| GpDSR7 GSP1 R |  | AGAAACGGGGTCACCGGCATTGAAG |
| GpDSR7 NGSP1 R |  | GCAGGTCCCATCATCACGCCAGGAA |
| GpDSR7 GSP2 F | ACCCGCTCCTTGAAACACACACACG |  |
| GpDSR7 NGSP2 F | TCTTGTGTGTTGGGTCTGCTTGCTG |  |
| GpDSR7 ORF F+R | ATGGAGCCTCGCAACCATGAA | CTAGTGGGCCTGTGGGTTAGACTGA |
| GpDSR7Δ *Bam*HⅠF +*Sal*Ⅰ(+His+TGA) R | TTGGATCCATGGAGCCTCGCAAC | TTGTCGACTCAGTGGTGGTGGTGGTGGTGTGCACCCGACCCCATT |
| GpDSR7^Δ^ 224S F+R | AAAGAAATCCTGCACACTGCCTCC | TGTGCAGGATTTCTTTTGAGGAGC |
| GpDSR7^Δ^ 242S F+R | CCCCTCTAGAGCTGCACTCCATCTT | AGCTCTAGAGGGGAGATGGCTCTG |
| GpDSR7^Δ^ H250Y F+R | TCTTTATACGCCAGAGCCATCTCCC | GGCGTATAAAGACTGTGCCCTCAA |
| GpDSR7 pENTR F+R | CACCATGGAGCCTCGCAACCATGAA | GTGGGCCTGTGGGTTAGACTGAGCCTG |
| GpDSR7-ΔTM pENTR R |  | TGCACCCGACCCCATTTGACCC |
| GpDSR7-TM pENTR F | CACCATGCTTGCAATATCCCTTCCCTTTTC |  |
