# Supplementary material for: GpDSR7, a Novel E3 Ubiquitin Ligase Gene in Grimmia pilifera Is Involved in Tolerance to Drought Stress in Arabidopsis
Source: PLoS One. 2016 May 26;11(5):e0155455. doi: 10.1371/journal.pone.0155455 (PMC4882056; doi:10.1371/journal.pone.0155455)
Supplement: S2 Table — (DOCX) [file pone.0155455.s002.docx]

**S2 table. Specific primers sequences for RT-PCR of *Grimmia pilifera.***

| Gene name | Forward primer | Reverse primer | PCR product size (bp) | Cycles |
| --- | --- | --- | --- | --- |
| *GpDSR1* | AGCATCATCAGCCGACATTTACCTA | GGCAAATGATGCTGAAAATTGAAAA | 142 | 23 |
| *GpDSR2* | TTTTCACTTTCATCTCTTTGGGTCA | GCAAATGCAAGATACATGGACTAGC | 213 | 23 |
| *GpDSR3* | AACGACCTTCCTTCCGCTGTTCCCT | AGCAACACAGTGCCCCTTCTGCAAA | 182 | 22 |
| *GpDSR7* | TTTGATGGTGGAAGCTGTGGAGAGT | TGCTTATTGGGAAATGGGCACTTCT | 158 | 27 |
| *GpDSR9* | TGCAATAAGCTGATCCGCCGTTAGT | GCTTTTCCAACGGTGACGCTATTCT | 109 | 25 |
| *GpDSR10* | GACGGGCGCTACTGGACCATGTGGA | GGCCTGACAACCAAGAACCCGCAGA | 173 | 25 |
| *GpDSR11* | CGGCAAACAAGGTGATTATTATGTG | CAATGCTAGTGCCTCAAGATGAATC | 214 | 29 |
| *GpDSR14* | GGGGACGTTGAGGAGGTTGAGGAGG | CCAGACGACTCATACGACCCCCACC | 225 | 28 |
| *GpDSR15* | TTAACAGTAGCAAACCAACGCGGCA | TGTACTCGCTCATTGCTGTCGCCTT | 241 | 29 |
| *GpDSR17* | CGTTGTCCCTGTGGTCGAGCTTGGT | GACTCACCGATCACCTGCATTCCCC | 234 | 29 |
| *GpDSR18* | TGCACCTGACACGAAAAGAATTTGA | CTGAGACAGAACGGTTGCTTTCGGA | 217 | 29 |
| *GpDSR19* | ACGCCAAAGCAATAGATACCGCTCC | TGCAGGACAGTCTTTTGGCCTCTTT | 194 | 30 |
| *GpDSR20* | TGCACCTCGCATCGCAGTCATACAA | GACCTCGATGCCGAAGACCTCCAAC | 299 | 26 |
| *GpDSR23* | ATACCGCAAGAACAGCTCCGCAACG | GCGGAGGTTTATGGGTGTTGAGGGG | 162 | 30 |
| *GpDSR24* | GCTCTTGTTGGCACTTGGGACTCTT | GCTATACTTCAGGCCAGGGCAGGTC | 233 | 30 |
| *GpDSR25* | GCTCTTTTCATTGATAACGCACGCT | CTGGCGGCATAATGTAAGTTGAGGT | 278 | 24 |
| *GpDSR26* | GCCTCGAACGAAGCAGTAAAACCCA | GCTTCACTGGAGGGTGGTTCTTTGG | 163 | 26 |
| *GpActin* | ACGTTGCCATTCAGGCTGTGCTATC | AGGGCAACGTAAGCCAGCTTCTCC | 264 | 28 |
