# Supplementary material for: GpDSR7, a Novel E3 Ubiquitin Ligase Gene in Grimmia pilifera Is Involved in Tolerance to Drought Stress in Arabidopsis
Source: PLoS One. 2016 May 26;11(5):e0155455. doi: 10.1371/journal.pone.0155455 (PMC4882056; doi:10.1371/journal.pone.0155455)
Supplement: S3 Table — (DOCX) [file pone.0155455.s003.docx]

**S3 table. Specific primers sequences for qRT-PCR expression analysis of drought stress-related genes in *Arabidopsis*.**

| Gene name | Forward primer | Reverse primer |
| --- | --- | --- |
| *ABI1* | GGAAGTGACGGCTGTGAAGAG | CTTTCCCTTCCTTTCTCCGCT |
| *ABF4* | TCGAGGACGAAGAAGCAATAC | TCTTCTGCATTTCCACCATTT |
| *ABI5* | GGCTAAGGGGAAGGAAAAGAG | TGTTGCTTCCTCTTCCTCTCC |
| *GPA1* | CTGTGATATTTTGTGCTGCCA | AATCTCTGAACCACTCGCAAA |
| *RD22* | CCACAAGGCGATGATGACGAC | GAGGAAGTGGCAGACCGGAAC |
| *RD29a* | AGATCAAACTCAAGTGGCGGG | CCGCCACATAATCTCTACCCG |
| *RD29b* | GACCAGATAGCGGAGGGGAAA | CCGAAAACCCCATAGTCCCAA |
| *P5CS1* | TTGCAGAGCTATTCCTTCGCC | AGCTTGGATGGGAATGTCCTG |
| *PLD1* | GTACGAACACCTGGGAATGCT | CTGATTTGGTGCCGAGGATAC |
| *GAPDH* | TGAAGGACTGGAGAGGTGGAAGAGC | GTTGTCGCCAACGAAGTCAGTTGAG |
