# Supplementary material for: GpDSR7, a Novel E3 Ubiquitin Ligase Gene in Grimmia pilifera Is Involved in Tolerance to Drought Stress in Arabidopsis
Source: PLoS One. 2016 May 26;11(5):e0155455. doi: 10.1371/journal.pone.0155455 (PMC4882056; doi:10.1371/journal.pone.0155455)
Supplement: S4 Table — (DOCX) [file pone.0155455.s004.docx]

**S4 table. Summary on screening results of forward library ESTs cloned by SSH.**

| Descriptive category |  |
| --- | --- |
| Total clones | 923 |
| Positive clones by PCR test | 803 |
| Number of efficient ESTs | 574 |
| Mean EST length (bp) | 529 |
| Number of singletons | 114 |
| Number of contiguous sequences (contig) | 126 |
| Number of unigenes | 240 |
| Number of ESTs in contigs | 460 |
| Contig EST redundancy (%) ^a^ | 80.14 |
| ^a^ Percentage of the ESTs assembled in the contigs/total number of ESTs. | |
